# Fitting of Growth Curves and Estimation of Genetic Relationship between Growth Parameters of Qianhua Mutton Merino

**DOI:** 10.3390/genes15030390

**Published:** 2024-03-21

**Authors:** Jiarong Li, Xuesong Shan, Yang Chen, Chongshun Xu, Lin Tang, Huaizhi Jiang

**Affiliations:** College of Animal Science and Technology, Jilin Agricultural University, Changchun 130118, China; lijiarong93@126.com (J.L.); shanxuesong@jlau.edu.cn (X.S.); chenyang7419@163.com (Y.C.); xuchongshun927@163.com (C.X.); tanglin6171113@163.com (L.T.)

**Keywords:** growth curve, genetic correlation, heritability, genetic parameters, Qianhua Mutton Merino

## Abstract

Qianhua Mutton Merino is a dual-purpose (meat and wool) breed of sheep that has been newly developed in China. In this study, we assessed the growth and development of the Qianhua Mutton Merino sheep breed under house feeding conditions by measuring the body weight and chest circumference of 2300 rams and ewes of this breed aged 0–24 months. Based on the fitting results of three nonlinear growth models, namely Logistic, Gompertz, and von Bertalanffy, in Qianhua Mutton Merino, we selected the von Bertalanffy model because of its highest fitting degree among all models (R^2^ > 0.977). The significant analysis of the combined fixation of each sheep body’s weight and bust took place (A: mature body weight, B: adjustment parameter, K: instant relative growth rate). The results revealed that parameters A, B, and K of body weight and chest circumference have high heritability and thus could be used as target traits for genetic improvement. Moreover, the correlation strength among A, B, and K suggested that these parameters can be used as a reference to adjust the genetic parameters in the growth model to genetically improve the body size of Qianhua Mutton Merino during breeding.

## 1. Introduction

Growth and development are vital traits of domestic meat animals. Although the growth and development of animals are controlled by genetic and environmental factors, the basic characteristics of the growth and development of a species or a breed of domestic animals are relatively stable.

Growth curves are commonly used to determine developmental characteristics and their cumulative changes, as well as to explore the relationship between the entire and a part of a livestock group during different growth stages. The fitting of growth curves of growth functions is an extremely effective tool for assessing different management factors for breeding purposes [1]. Growth functions have been used extensively to represent changes in size with age, which in turn facilitates the evaluation of the genetic potential of animals for growth and matching nutrition to possible growth [2]. Commonly used growth curve models include the Logistic, Gompertz, Brody, von Bertalanffy, and Richands models [1,2,3,4,5]. The correlation strength among variables estimated through different models has been shown to be high. Generally, the goodness of fit of these models for growth data for various species tends to be similar to those of the Logistic, Gompertz, and von Bertalanffy models [4]. Growth curve parameters serve as potentially useful criteria for determining the relationship between weight and age through parameter selection, and the selection of the expected value of growth curve parameters can be useful in obtaining an optimal growth curve [4,6]. Typically, growth curve parameters are estimated using nonlinear mathematical functions that enable data summarization from a large number of longitudinal (weight–age) data of each individual [7]. Pala demonstrated that growth rate is related to maturity rate and mature body weight, which in turn are related to the lifetime productivity parameters of an animal [8].

Furthermore, the posterior distribution of parameter values could be generated using the Gibbs sampling algorithm [9,10,11], and a random sample of parameter estimation could be estimated according to a provided data set, which is proportional to the product of parameter probability and observation probability [12,13]. Moreover, the restricted maximum likelihood (REML) and Bayesian methods are widely used in animal breeding to estimate genetic parameters and variance components [14,15]. Carneiro reported that the Bayesian method is useful in analyzing small groups when a large amount of historical data is available [16]. Furthermore, some scholars have proposed that sheep have a large body size that leads to better productivity [17]; however, most studies on the animal growth curve have considered animals aged between 0 and 12 months, and only a few studies have reported the simulation of the growth curve of sheep and goats aged between 12 and 24 months.

Qianhua Mutton Merino is a newly developed, dual-purpose (meat and wool) breed of sheep that was obtained through the cross-breeding of the introduced South African Mutton Merino with the domestic Northeast China fine-fleece sheep in China in 2018 [18]. This newly developed sheep breed dwells in the pastoral farming zone, with an over 130% lambing rate, and possesses characteristics such as strong adaptability, large body weight, rapid growth and development, high meat yield, homogeneous hair, and 66s hair as the main body. As a newly bred meat sheep breed, the exploration of the genetic characteristics of Qianhua Mutton Merino’s growth and development could reveal its germplasm characteristics, and it also may serve as a reference for improving the breeding of this sheep breed. Therefore, this study investigated the genetic relationship between the growth curve parameters of Qianhua Mutton Merino.

## 2. Materials and Methods

Among the core breeding group of Qianhua Mutton Merino raised by Jilin Qian’an Zhihua Breeding Sheep Breeding Co., Ltd. (Qianan, China), 896 rams and 1404 ewes born between 10 January and 13 January 2018 and exhibiting good physical condition, appetite, and health status were randomly selected and studied until 20 January 2020. All the sheep were fed in-house, and all the experimental sheep were fed diets as per the NRC (2007) standard. The sheep were vaccinated regularly and dewormed timely. The birth weight of lambs was measured before colostrum sucking within 1 h of birth, and the body weight during other periods was measured as the fasting weight before the morning feed. The body weight and bust circumference of the experimental sheep were continuously measured at 3, 6, 12, 18, and 24 months of age until January 2020.

In this study, three nonlinear models, namely, Logistics, Gompertz, and von Bertalanffy, were selected for the fitting of the growth curve. Table 1 presents the formula and characteristics of the fitting models. In each model, W_t_ represents the estimation of body weight and bust circumference at ‘t’ months of age. Notably, parameter A indicates the mature weight (chest circumference); K indicates the instantaneous relative growth rate of body weight (chest circumference) relative to mature body weight (chest circumference), indicating the speed at which the animal approaches adult body weight (chest circumference); B indicates the adjustment parameter related to the initial body weight (chest circumference), determined using the initial values of Wt and ‘t’; ‘t’ indicates the age in months; and ‘w’ indicates the body weight at the inflection point (chest circumference).

The daily growth in body size is given as follows: $G=(W_{t}-W_{0})/t$, wherein W_t_ is the subsequent body size value, W_0_ is the previously measured body size value, and ‘t’ is the number of days.

The relative growth rate is given as follows: $R=2\times (W_{t}-W_{0})/\left(W_{t}+W_{0}\right)\times 100\%$.

The fitting degree formula is as follows: $R^{2}=\sum (W-W_{p}{)}^{2}/(W-W_{m}{)}^{2}$.

Absolute growth indicates the absolute speed of livestock growth and development during a certain period, and this study assessed the daily growth to ascertain the absolute growth of the body weight and chest circumference of Qianhua Mutton Merino. By contrast, relative growth indicates the intensity of livestock growth and development. The fitting degree R^2^ was used to evaluate the growth curve model, wherein WP denotes the predicted average value and Wm denotes the actual average value. Notably, the closer the R^2^ is to 1, the better the fitting degree of growth and development is and the closer it is to its growth and development. In addition, we used the Gibbs sampling algorithm to conduct Bayes estimation for the variance and genetic parameters of the weight and chest circumference growth curves of Qianhua Mutton Merino, which will be used to design future programs involving Qianhua Mutton Merino and will provide a reference for further breeding and the scientific breeding of Qianhua Mutton Merino.

The General Linear Model (GLM) program of SPSS 25.0 (IBM) was used for the significance analysis of data combined with fixed effects, and the significance level of the fixed effect in the model was *p* < 0.05. Fixed effects in the model formulas of parameters A, B, and K included sex, age, and population, while the random effects were the random additive genetic effects of animals [17]. The univariate animal model suitable for the genetic analysis of growth curve parameters is as follows:

(1) Y=Xβ+Zu+e

where ‘Y’ is the observed value vector of all traits; X is the structure matrix of fixed effects; β is the fixed-effect vector, including gender, age, etc.; Z is the structure matrix of random effects; ‘u’ is the individual additive-effect vector; and ‘e’ is the random residual-effect vector. Blup90 software was used to analyze the variance and genetic parameters.

The narrow-sense heritability of trait a was calculated as

(2) $$h_{a_{i}}^{2}=\frac{\sigma_{a_{i}}^{2}}{\sigma_{a_{i}}^{2}+\sigma_{e_{i}}^{2}}$$

where $\sigma_{a_{i}}^{2}$ and $\sigma_{e_{i}}^{2}$ are the estimated additive genetic variance and the estimated residual variance for trait i.

Genetic correlation between trait i and trait j was calculated as

(3) $$r_{a_{\mathrm{ij}}}=\frac{\sigma_{a_{\mathrm{ij}}}}{\sqrt{\sigma_{a_{i}}^{2}\cdot \sigma_{a_{j}}^{2}}}$$

where $\sigma_{a_{\mathrm{ij}}}$ is the estimated additive genetic covariance between trait i and trait j, $\sigma_{a_{i}}^{2}$ is the estimated additive genetic variance for trait i, and $\sigma_{a_{j}}^{2}$ is the estimated additive genetic variance for trait j.

Residual correlation between trait i and trait j was calculated as

(4) $$r_{e_{\mathrm{ij}}}=\frac{\sigma_{e_{\mathrm{ij}}}}{\sqrt{\sigma_{e_{i}}^{2}\cdot \sigma_{e_{j}}^{2}}}$$

where $\sigma_{e_{\mathrm{ij}}}$ is the estimated additive residual correlation between trait i and trait j, $\sigma_{e_{i}}^{2}$ is the estimated additive residual variance for trait i, and $\sigma_{e_{j}}^{2}$ is the estimated additive residual variance for trait j.

Phenotypic correlation between trait i and trait j was calculated as

(5) $$r_{p_{\mathrm{ij}}}=\frac{\sigma_{a_{\mathrm{ij}}}+\sigma_{e_{ij}}}{\sqrt{\left(\sigma_{a_{i}}^{2}+\sigma_{e_{i}}^{2})\cdot (\sigma_{a_{j}}^{2}+\sigma_{e_{j}}^{2}\right)}}$$

According to the Bayes formula, if the prior probability of additive direct effects has a multivariate normal distribution, the mean is 0, the variance is σ_a_^2^, and σ_a_^2^ is the additive direct genetic variance; if the residual effect (posterior probability) has a multivariate normal distribution, the mean is 0, and the variance is 1_n_ σ_a_^2^, where the order of the identity matrix 1n is equal to the number of individual records and σ_a_^2^ is the residual. A contrasting analysis was conducted for the three variables, wherein the model that fitted for each feature in the univariate analysis was used. If the genetic and residual (covariance) variance matrices followed the inverse Wishart distribution, the GIBBS1F90 of Blup90 (Ignacy Misztal) program was used to estimate the marginal posterior distribution of the parameters and variance components. The GIBBS1F90 sampler was run 300,000 times, and the first 60,000 runs were discarded as the aging period [19]. Then, the mean, SD, and 95% high posterior density (HPD) interval of all parameters were calculated for each marginal progeny.

## 3. Results and Discussion

### 3.1. Growth Curve

The Logistic, Gompertz, and von Bertalanffy models were selected for the nonlinear fitting of the growth curve to the body weight and chest circumference of Qianhua Mutton Merino at birth and at 3, 6, 12, 18, and 24 months of age. The fitting parameters were estimated; the fitting degree (R^2^) and the inflection age and inflection body weight (chest circumference) were calculated based on the three models, and the results are cited in Table 2.

We observed that the Logistic, Gompertz, and von Bertalanffy models displayed a high fitting degree for the body weight (R^2^ > 0.95) and chest circumference (R^2^ > 0.97) of Qianhua Mutton Merino. Of these models, the fitting degree of the von Bertalanffy model concerning the body weight (R^2^ > 0.979) was found to be superior to those of the Logistic (R^2^ > 0.957) and Gompertz (R^2^ > 0.973) models. The estimated inflection point age of rams and ewes estimated using the von Bertalanffy model was 5.02 and 1.80 months, respectively; the inflection point weight was 41.66 and 18.75 kg, respectively; and the maximum daily growth rate was 220.80 and 253.13 g/d, respectively. Notably, the inflection point month of ewe weight was 3.22 months earlier than that of ram. The fitting degree of the von Bertalanffy (R^2^ > 0.977) model regarding the chest circumference of rams and ewes was also superior to those of the Logistic (R^2^ > 0.970) and Gompertz (R^2^ > 0.975) models. The inflection point age of rams and ewes of Qianhua Mutton Merino was 0.17 and 0.01 months, respectively; the inflection point chest circumference was 39.52 and 36.37 cm, respectively; and the maximum daily growth was 3.656 and 3.564 mm/d, respectively. Notably, the inflection point month of ewe weight was 0.16 months earlier than that of ram. This result is consistent with those reported by [20,21] Topal et al. (2004) and Ullah et al. (2013) (Figures S1 and S2).

As animal growth is a cumulative process, absolute and relative growth rates can effectively describe the growth process and its efficiency. Absolute growth serves as a basis for assessing an animal’s nutritional status and determining whether its growth and development are normal. Figures S2 and S3 demonstrate that the weights of both male and female Qianhua Meat-type Merino sheep reach peak absolute and relative growth rates between birth and three months of age. These findings align with domestic research on Hubei black-headed sheep [22], Gansu Alpine Fine Wool Sheep [23], Hu sheep [24], and Tibetan sheep [25]. The results of the absolute and relative growth curves suggest that the rapid growth rate prior to weaning is directly related to breast milk, while the decrease in relative growth rate after weaning is attributed to changes in nutritional supply and living environment (Figures S1 and S2). Therefore, it is recommended to strengthen early feeding management, especially by introducing solid food early, to meet the nutritional demands of Qianhua Meat-type Merino sheep during their growth and development. Additionally, Figure S2 shows that the peak of the actual weight curve occurs between birth and three months of age, while the fitted curve indicates peak weights at 9–12 months for male sheep and 3–6 months for female sheep. This suggests that both male and female Qianhua Meat-type Merino sheep experience a period of rapid weight gain between birth and 12 months and birth and 6 months, respectively. The deviation between the actual and fitted curves may be due to the relatively high growth rates during the first 6 months, particularly the first 3 months, combined with relatively long measurement intervals. Therefore, close monitoring during this critical stage is essential in subsequent feeding management.

The actual body weight and body size of rams and ewes of Qianhua Mutton Merino were consistent with the trends of the theoretical values of growth curves fitted by the three models (as shown in Figures S1–S6), and the relative growth rate had a high degree of coincidence. According to the growth curves fitted by the three models, both the absolute and relative growth curves revealed that the growth peak of ewes is reached between 0 and 3 months of age, whereas that of rams is reached between 3 and 6 months of age and 9 and 12 months of age, with the growth rate exhibiting a downward trend after 12 months, particularly after 18 months.

### 3.2. Genetic Relationship between the Parameters

Based on the experimental results, the von Bertalanffy model was selected to estimate the growth curve parameters of the body weight and chest circumference of each Qianhua Mutton Merino sheep. The data beyond the range of the normal growth curve were excluded for each parameter, and subsequent calculations were performed (Table 3 and Table 4).

The estimation of variance components and the direct heritability of growth curve parameters revealed no or a slight difference between the median, mode, and mean, indicating that direct heritability has a normal posterior distribution (Supplementary Figures S1 and S2). The high heritability of the mature body weight might be attributed to the weak effects of environmental factors on Qianhua Mutton Merino. Generally, factors such as animal species, genetic variation within the population, management and environmental conditions, and methods of parameter estimation affect the estimated values [12].

The direct genetic correlation coefficient between A and K in the body weight growth curve was −0.53, revealing a moderate negative correlation (Table 5, Supplementary Figure S3), which indicated that the growth and development rates were high; the smaller the K, the higher the mature weight of Qianhua Mutton Merino, and in practice, the maturity weight can be increased by reducing the growth rate. Furthermore, the direct genetic correlation coefficient between A and B was −0.24, revealing a weak negative correlation, which indicated that despite the effect of multiple births of Qianhua Mutton Merino on the birth weight of lambs, the late growth rate is fast, and the low growth rate caused by a double tire can be effectively compensated. The direct genetic correlation coefficient between B and K was 0.40, revealing a moderate positive correlation, thereby indicating that the rehearsal is low, but the growth rate is still very high. The results are consistent with those of Abegaz [26], Bathaei [6], Malhado [27], and Stobart [28]. Farhat [29] used five models (Gompertz, Logistic, Negative Exponential, Brody, and Bertalanffy) to fit the weight of Harnai Sheep and determined the correlation between parameters in the best fit model. They reported a negative correlation between A and K and a positive correlation between B and K. This finding is consistent with that of the present study. Lambe [5] used the Brody function to fit the growth curve at birth, as well as at 3, 6, 12, 18, and 24 months of age for Ethiopischen Horro sheep and estimated the genetic correlation among A, B, and K. A and K were found to have medium positive genetic correlations with weight at 6 and 12 months of age, indicating that these traits can be used to indirectly select the growth curve parameters.

The direct genetic correlation between A and K in the chest circumference growth curve was −0.83, revealing a strong negative correlation (Table 6, Supplementary Figure S4) and indicating that for the growth and development speed, the smaller the K, the larger the chest circumference is, thereby demonstrating that the increase in body weight is mainly reflected in the change in chest depth. The direct genetic correlation coefficient between A and B was 0.27, revealing a weak correlation and indicating that despite the low birth weight, the sheep had an excellent meat body size. The direct genetic correlation coefficient between B and K was 0.03, indicating that the size of the chest circumference at birth does not affect the subsequent growth rate of the chest circumference. Taken together, the results indicated that Qianhua Mutton Merino possesses a strong fattening ability, strong weight gain ability, and excellent meat body size.

## 4. Conclusions

In this study, three models were used to fit the growth curves of two phenotypic traits, namely, the body weight and chest circumference of Qianhua Mutton Merino, and a suitable growth model for determining breed characteristics was obtained. The genetic parameters of the von Bertalanffy growth curve of body weight and chest circumference were estimated on the basis of curve simulation. The results indicated that Qianhua Mutton Merino has a strong weight growth ability and excellent meat body size. The study also suggests that the breeding strategy of Qianhua Mutton Merino can be improved by changing the K value (i.e., the instantaneous growth rate) in the curve model. The traditional breeding of mutton sheep considers the body weight, chest circumference, and weight gain individually or involves a static comprehensive evaluation method; however, a dynamic comprehensive evaluation method for estimating the meat body size and fattening potential has not been reported. The results revealed that selecting the comprehensive body size of sheep by using the growth model parameters is an excellent strategy. Therefore, the growth potential of livestock during different stages of growth can be analyzed using the biological significance of the parameters in the growth curve model and the relationship between them, facilitating scientific and reasonable breeding programs and feeding management regulations. This is of great significance for guiding breeding practice and scientific feeding, thereby ensuring appropriate weight gain, achieving ideal weight, and improving the production level and production efficiency.

## Figures and Tables

**Table 1:** Formula and related parameters of Logistic, Gompertz, and von Bertalanffy growth curve models.

Model	Expression	Inflection Point Weight/kg	Inflection Point Months	Maximum Daily Gain/g∙d^−1^
Logistic	W_t_ = A/(1 + Be^−Kt^)	A/2	(LnB)/K	wk/2
Gompertz	W_t_ = Ae^−Bexp(−Kt)^	A/e	(LnB)/K	Wk
von Bertalanffy	W_t_ = A(1 − Be^−kt^)^3^	8A/27	(Ln3B)/K	3kw/2

Note: A: the mature weight (chest circumference); K: the instantaneous relative growth rate of body weight (chest circumference) relative to mature body weight (chest circumference), indicating the speed at which the animal approaches adult body weight (chest circumference); B: the adjustment parameter related to the initial body weight (chest circumference); t: the age in months; w: the body weight at the inflection point (chest circumference); Wt: estimated weight (chest circumference) at t months of age.

**Table 2:** The parameter-estimated value and fitting degree of three growth curve models of Qianhua Mutton Merino.

Phenotypic Traits	Model	Gender	A	B	K	R^2^	Inflection Point Months	Inflection Point Weight/kg	Maximum Daily Gain/g∙d^−1^
Weight	Logistic	Ram	127.156	6.39	0.207	0.973	8.96	63.58	219.35
		Ewe	61.477	6.096	0.499	0.957	3.62	30.74	255.65
	Gompertz	Ram	135.048	2.317	0.132	0.984	6.37	49.68	218.59
		Ewe	62.58	2.209	0.325	0.973	2.44	23.02	249.38
	Von Bertalanffy	Ram	140.617	0.567	0.106	0.989	5.02	41.66	220.80
		Ewe	63.284	0.542	0.27	0.979	1.80	18.75	253.13
chest circumference	Logistic	Ram	130.655	2.219	0.278	0.979	2.87	65.33	3.027
		Ewe	120.825	2.102	0.287	0.970	2.59	60.41	2.890
	Gompertz	Ram	131.806	1.296	0.22	0.982	1.18	48.49	3.556
		Ewe	122.09	1.195	0.218	0.975	0.82	44.91	3.263
	Von Bertalanffy	Ram	133.387	0.344	0.185	0.986	0.17	39.52	3.656
		Ewe	122.753	0.334	0.196	0.977	0.01	36.37	3.564

Note: A: the mature weight (chest circumference); K: the instantaneous relative growth rate of body weight (chest circumference) relative to mature body weight (chest circumference), indicating the speed at which the animal approaches adult body weight (chest circumference); B: the adjustment parameter related to the initial body weight (chest circumference); R^2^: degree of fitting.

**Table 3:** The posterior mean, median, and mode of genetic and residual variance between growth curve parameters of weight in Qianhua Mutton Merino (standard deviations [PSD] of marginal posterior distribution of mean estimates are in brackets).

Parameter	Item	Mean	Median	Mode	HPD
A	$\sigma_{a}^{2}$	5.54 (15.61)	5.00	10.14	0.41–12.51
	$\sigma_{e}^{2}$	3.34 (4.16)	3.31	3.90	0.30–6.77
	$\sigma_{p}^{2}$	8.88 (7.74)	8.46	7.40	5.19–13.92
	$h_{a}^{2}$	0.56 (0.09)	0.60	0.18	0.07–0.97
B	$\sigma_{a}^{2}$	0.00036 (0)	0.00035	0.00041	0.000098–0.00067
	$\sigma_{e}^{2}$	0.00016 (0)	0.00016	0.0002	0.000038–0.00033
	$\sigma_{p}^{2}$	0.00053 (0)	0.0005	0.00047	0.00033–0.0008
	$h_{a}^{2}$	0.66 (0.04)	0.69	0.67	0.26–0.94
K	$\sigma_{a}^{2}$	0.00066 (0)	0.00062	0.0011	0.00022–0.0012
	$\sigma_{e}^{2}$	0.00037 (0)	0.00035	0.0003	0.00014–0.00068
	$\sigma_{p}^{2}$	0.001 (0)	0.001	0.0008	0.00065–0.0016
	$h_{a}^{2}$	0.62 (0.03)	0.64	0.91	0.29–0.88

Note: $h_{a}^{2}$ = Direct heritability; $\sigma_{a}^{2}$ = Direct additive genetic variance; $\sigma_{e}^{2}$ = Residual variance; $\sigma_{p}^{2}$ = Phenotype variance; HPD = 95% high posterior density interval.

**Table 4:** The posterior mean, median, and mode of genetic and residual variance between growth curve parameters of chest circumference in Qianhua Mutton Merino (standard deviations [PSD] of marginal posterior distribution of mean estimates are in brackets).

Parameter	Item	Mean	Median	Mode	HPD
A	$\sigma_{a}^{2}$	19.40 (161.94	17.99	18.09	1.98–41.86
	$\sigma_{e}^{2}$	11.41 (42.76)	11.19	14.46	1.73–22.53
	$\sigma_{p}^{2}$	30.81 (91.27)	29.48	25.39	17.82–48.35
	$h_{a}^{2}$	0.58 (0.07)	0.61	0.5	0.09–0.95
B	$\sigma_{a}^{2}$	0.00012 (0)	0.00011	0.00012	0.00003–0.00023
	$\sigma_{e}^{2}$	0.00006 (0)	0.000058	0.0001	0.000016–0.00012
	$\sigma_{p}^{2}$	0.00018 (0)	0.00017	0.00017	0.00011–0.00027
	$h_{a}^{2}$	0.63 (0.04)	0.66	0.79	0.24–0.92
K	$\sigma_{a}^{2}$	0.00032 (0)	0.0003	0.0002	0.00011–0.0006
	$\sigma_{e}^{2}$	0.00018 (0)	0.00017	0.00013	0.00007–0.00032
	$\sigma_{p}^{2}$	0.0005 (0)	0.00048	0.00038	0.00031–0.00075
	$h_{a}^{2}$	0.62 (0.03)	0.63	0.50	0.30–0.88

$h_{a}^{2}$ = Direct heritability; $\sigma_{a}^{2}$ = Direct additive genetic variance; $\sigma_{e}^{2}$ = Residual variance; $\sigma_{p}^{2}$ = Phenotype variance; HPD = 95% high posterior density interval.

**Table 5:** The posterior mean, median, and mode of genetic, residual, and phenotypic correlations between growth curve parameters of weight in Qianhua Mutton Merino (standard deviations [PSD] of marginal posterior distribution of mean estimates are in brackets).

Correlation	Item	Traits		
		A-B	A-K	B-K
r_a1a2_	Mean	−0.24 (0.35)	−0.53 (0.23)	0.40 (0.13)
	Median	−0.34	−0.71	0.47
	Mode	−1.00	−1.00	0.83
	HPD	−0.99–0.87	−0.99–0.56	−0.31–0.87
r_e1e2_	Mean	−0.23 (0.39)	−0.53 (0.20)	0.47 (0.11)
	Median	−0.34	−0.66	0.53
	Mode	0.97	−1.00	−0.90
	HPD	−0.99–0.95	−0.98–0.58	−0.17–0.90
r_p1p2_	Mean	−0.26 (0.04)	−0.60 (0.02)	0.45 (0.02)
	Median	−0.28	−0.62	0.46
	Mode	0	−0.46	0.45
	HPD	−0.57–0.09	−0.80–−0.34	0.17–0.68

r_a1a2_ = direct genetic correlation between growth curve parameters; r_e1e2_ = residual correlation between growth curve parameters; r_p1p2_ = phenotypic correlation between growth curve parameters; HPD = highest posterior density at 95%.

**Table 6:** The posterior mean, median, and mode of genetic, residual, and phenotypic correlations between growth curve parameters of chest circumference in Qianhua Mutton Merino (standard deviations [PSD] of marginal posterior distribution of mean estimates are in brackets).

Correlation	Item	Traits		
		A-B	A-K	B-K
r_a1a2_	Mean	0.27 (0.25)	−0.83 (0.05)	0.03 (0.18)
	Median	0.36	−0.90	0.038
	Mode	0.39	−0.95	0.17
	HPD	−0.75–0.94	−1.00–−0.42	−0.70–0.73
r_e1e2_	Mean	0.28 (0.24)	−0.83 (0.04)	0.046 (0.18)
	Median	0.37	−0.88	0.043
	Mode	−1.00	−1.00	−0.99
	HPD	−0.78–0.93	−0.99–−0.46	−0.65–0.75
r_p1p2_	Mean	0.31 (0.03)	−0.84 (0.004)	0.033 (0.038)
	Median	0.32	−0.85	0.032
	Mode	0.31	−0.93	0.00
	HPD	−0.01–0.59	−0.92–−0.72	−0.29–0.35

r_a1a2_ = direct genetic correlation between growth curve parameters; r_e1e2_ = residual correlation between growth curve parameters; r_p1p2_ = phenotypic correlation between growth curve parameters; HPD = highest posterior density at 95%.

## Data Availability

The data presented in this study are available in the article and its Supplementary Materials.

## Supplementary Materials

The following supporting information can be downloaded at: https://www.mdpi.com/article/10.3390/genes15030390/s1, Figure S1: Cumulative growth curve of measured body weight and estimated weight of rams and ewes according to the models; Figure S2: Absolute growth curve of measured body weight and estimated weight of rams and ewes according to the models; Figure S3: Relative growth curve of measured body weight and estimated weight of rams and ewes according to the models; Figure S4: Cumulative growth curve of measured chest circumference and estimated chest circumference of rams and ewes according to the models; Figure S5: Absolute growth curve of measured chest circumference and estimated chest circumference of rams and ewes according to the models; Figure S6: Relative growth curve of measured chest circumference and estimated chest circumference of rams and ewes according to the models; Figure S7: Posterior distribution of the direct heritabilities for A, B, and K growth curve parameters of weight; Figure S8: Posterior distribution of the direct heritabilities for A, B, and K growth curve parameters of chest circumference; Figure S9: Posterior distribution of direct genetic correlations between growth curve parameters of weight (A-B, A-K, and B-K); Figure S10: Posterior distribution of direct genetic correlations b.
