# Supplementary material for: Fitting of Growth Curves and Estimation of Genetic Relationship between Growth Parameters of Qianhua Mutton Merino
Source: Genes (Basel). 2024 Mar 21;15(3):390. doi: 10.3390/genes15030390 (PMC10969850; doi:10.3390/genes15030390)
Supplement: Supplementary file 1 [file genes-15-00390-s001.zip › genes-2901010-supplementary.pdf]

Supplementary material

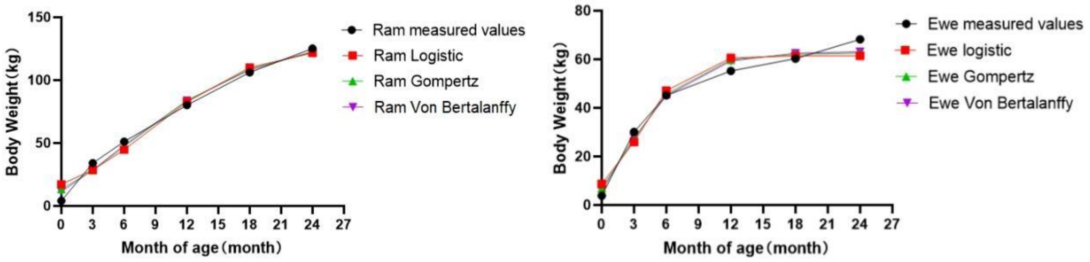

Figure S1. Cumulative growth curve of measured body weight and estimated weight of rams and ewes according to the models.

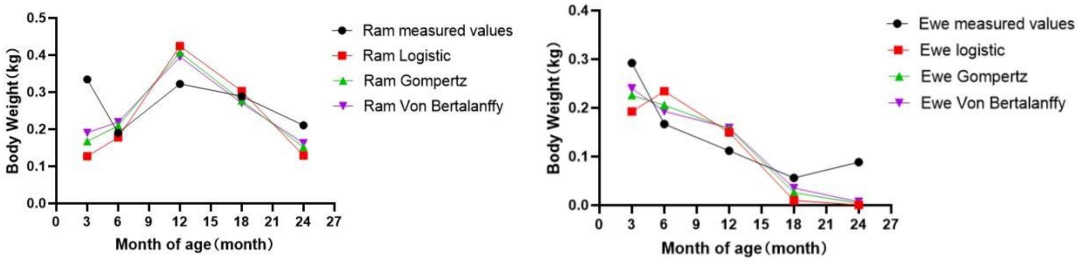

Figure S2. Absolute growth curve of measured body weight and estimated weight of rams and ewes according to the models.

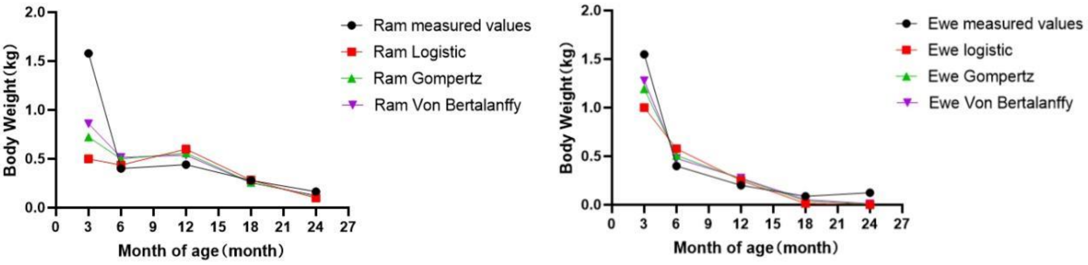

Figure S3. Relative growth curve of measured body weight and estimated weight of rams and ewes according to the models**.**

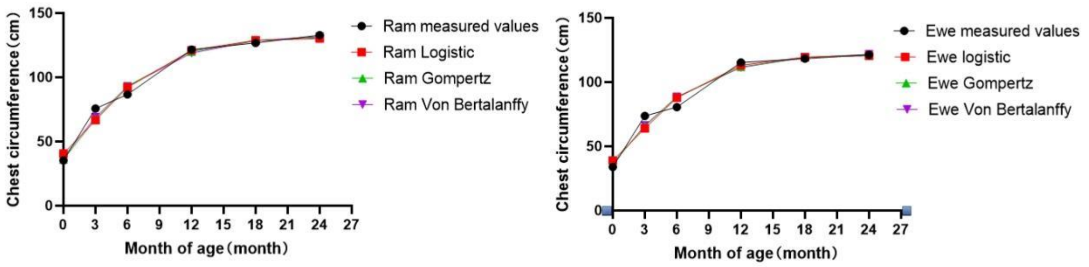

Figure S4. Cumulative growth curve of measured chest circumference and estimated chest circumference of rams and ewes according to the models.

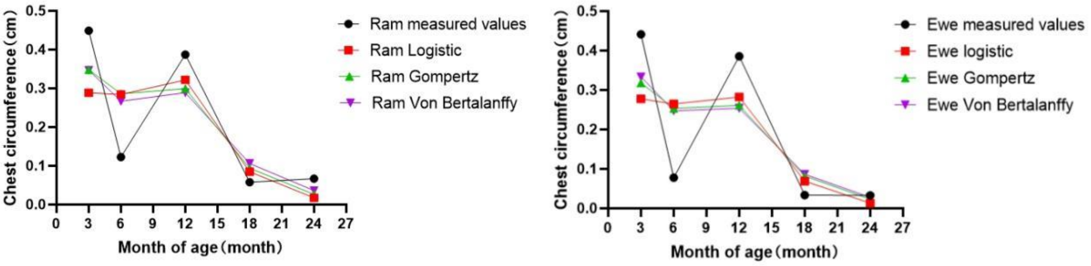

Figure S5. Absolute growth curve of measured chest circumference and estimated chest circumference of rams and ewes according to the models.

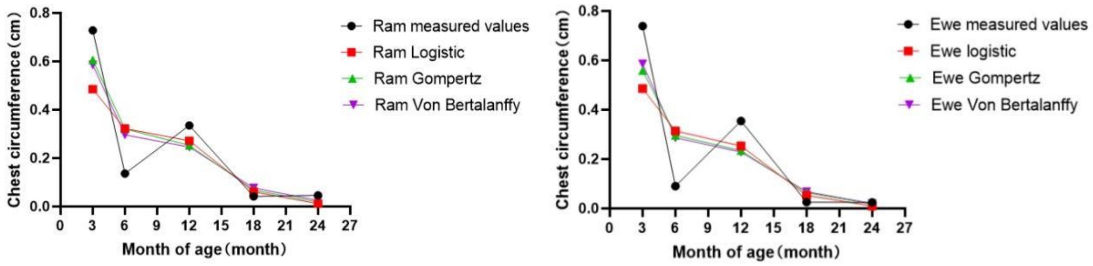

Figure S6. Relative growth curve of measured chest circumference and estimated chest circumference of rams and ewes according to the models.

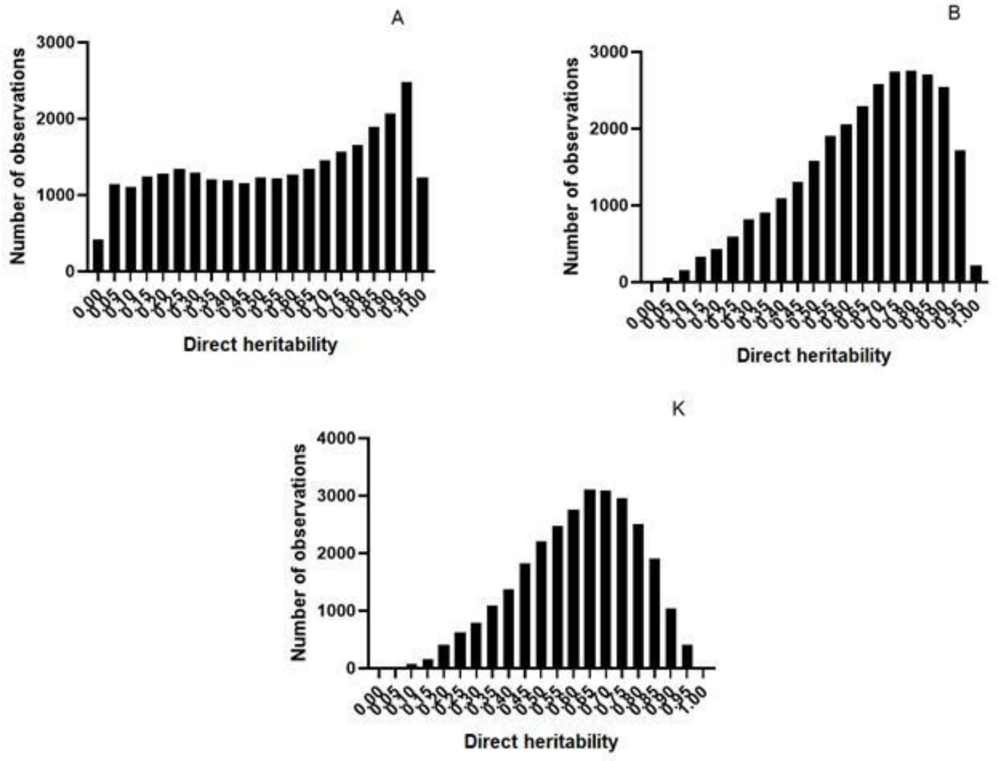

Figure S7. Posterior distribution of the direct heritabilities for A, B, and K growth curve parameters of weight.

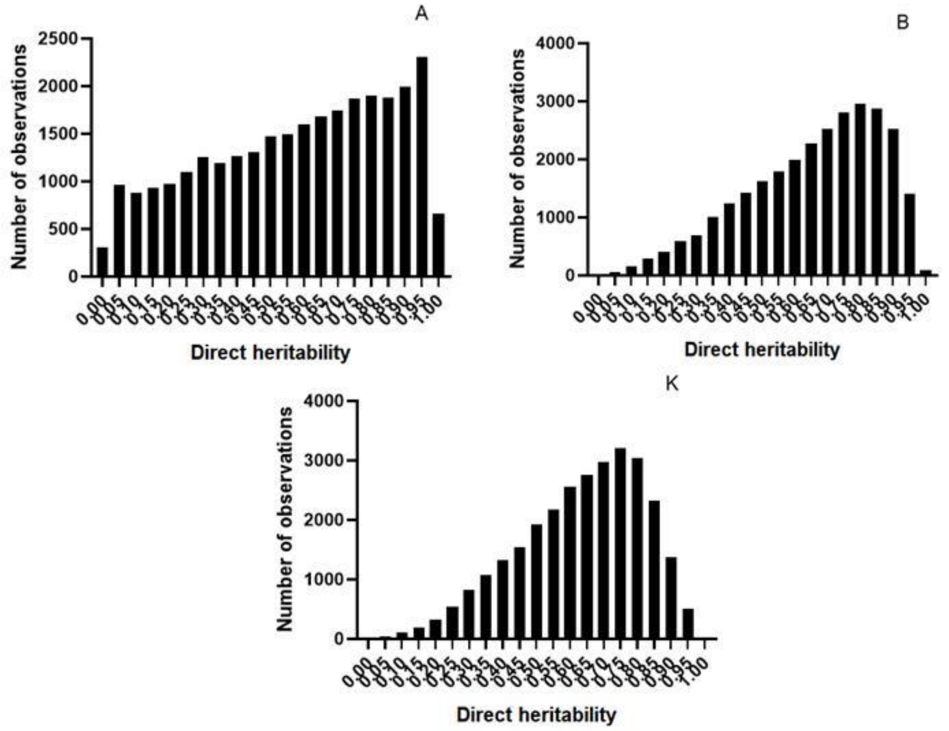

Figure S8. Posterior distribution of the direct heritabilities for A, B, and K growth curve parameters of chest circumference.

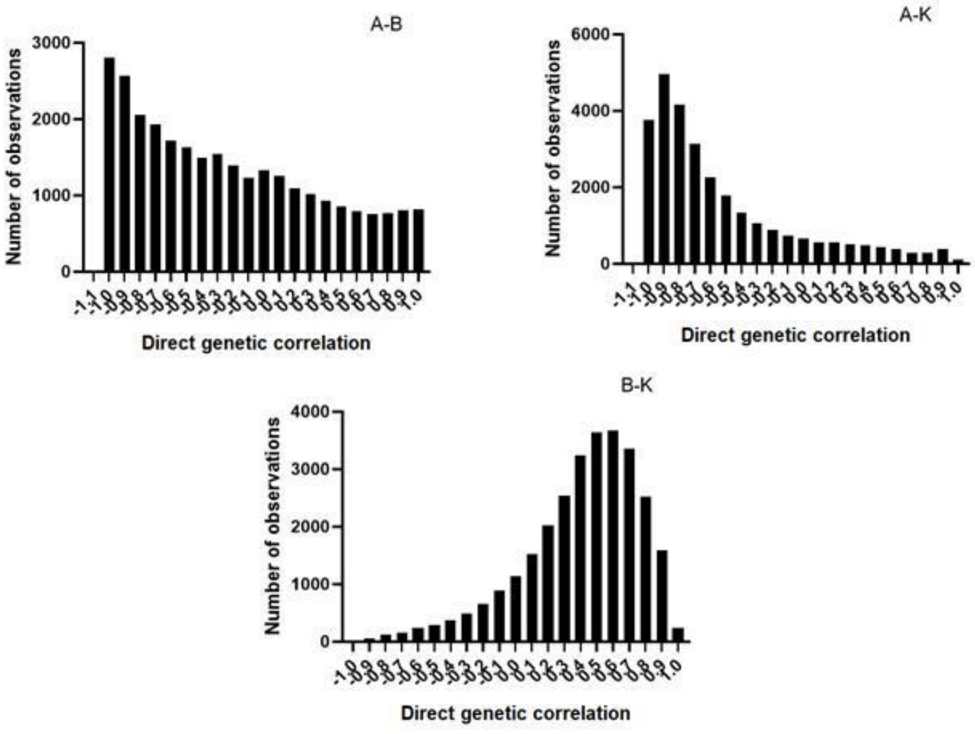

Figure S9. Posterior distribution of direct genetic correlations between growth curve parameters of weight (A-B, A-K, and B-K).

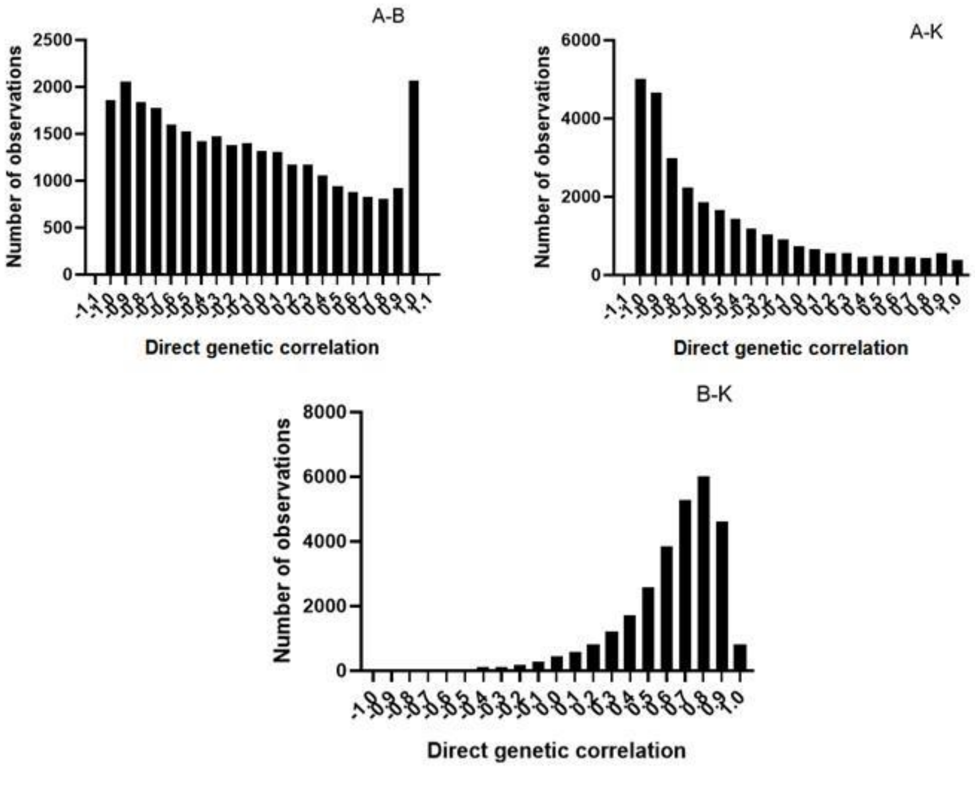

Figure S10. Posterior distribution of direct genetic correlations b
